# Supplementary material for: The Flavonoid Cyanidin Shows Immunomodulatory and Broad-Spectrum Antiviral Properties, Including SARS-CoV-2
Source: Viruses. 2023 Apr 18;15(4):989. doi: 10.3390/v15040989 (PMC10143848; doi:10.3390/v15040989)
Supplement: Supplementary file 1 [file viruses-15-00989-s001.zip › viruses-2316255-supplementary.pdf]

Supplementary Figure S1

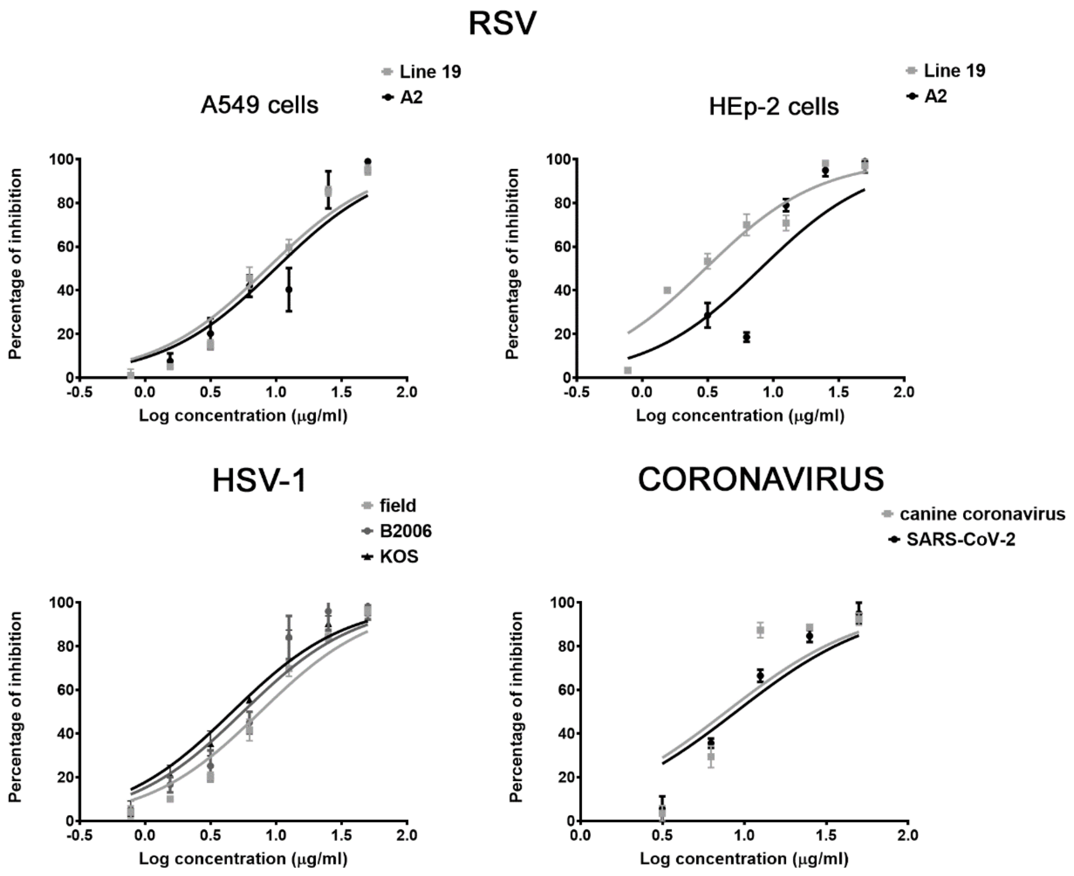

Supplementary Figure S1. Dose-dependent response of A18 on viral replication. Antiviral activity of different concentrations of A18 against RSV strains A2 and line 19 in HEp-2 and A549 cells; HSV-1 KOS, field and B2006 in HCLE cells; and SARS-CoV-2 in calu-3 cells, and canine coronavirus in CRFK cells. Data represent mean ± SD for n = 3 independent experiments, performed in triplicate.

Supplementary Figure S2

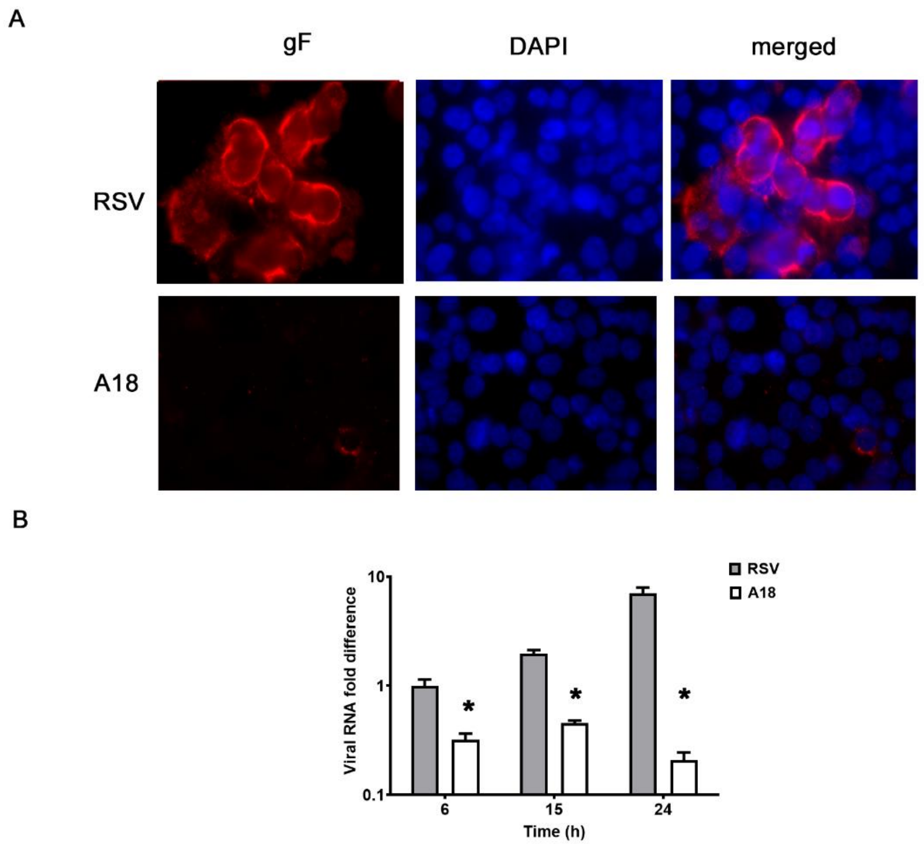

Supplementary Figure S2. Effect of A18 on RSV macromolecular synthesis in HEp-2 cells**.** (A) Protein expression. IF staining was performed to detect the intracellular localization RSV gF in HEp-2 cells infected with RSV A2 (moi= 1) and treated or not with A18 (25 μg/ml) at 24 h p.i. Magnification: 400X. (B) Viral RNA synthesis. Total cellular RNA was extracted at the indicated times p.i. in HEp-2 cells and viral RNA was quantified by qRT-PCR. The amounts of viral RNA in untreated and treated cells with A18 (25 μg/ml) in HEp-2 cells were calculated in comparison to the content of viral RNA in untreated infected cells at 6 h p.i., defined as 1. Data represent mean ±

SD for n = 3 independent experiments, performed in duplicate. *Significantly different from CV (p-value <0.05).

Supplementary Figure S3

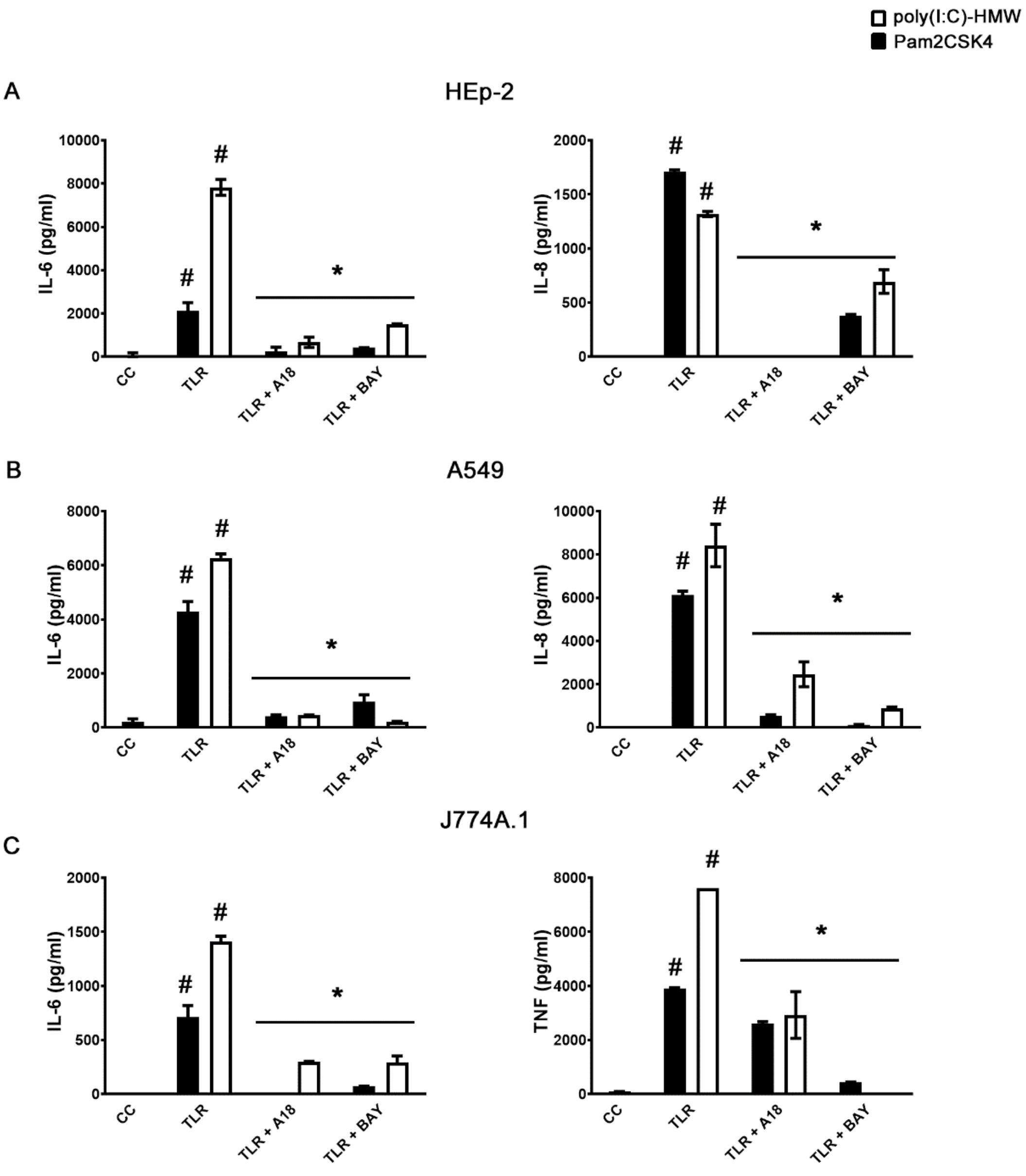

Supplementary Figure S3. Effect of A18 on cytokine production in TLRs stimulated cells. HEp-2 (A), A549 (B) and J774A.1 (C) cells were stimulated with Toll Like Receptor (TLR)2/6 (100 ng/ml) and TLR3 (10 μg/ml) ligands and treated or not with

A18 (25 μg/ml) and BAY 11-7085 (10 μg/ml) for 8 h. IL-6, IL-8 and TNF-α were determined by ELISA. CC: cell control (unstimulated cells). Data represent mean ± SD for n = 3 independent experiments, performed in duplicate. *Significantly different from TLRs stimulated cells; # Significantly different from CC (p-value <0.05).
